# Health care workers and disaster preparedness: barriers to and facilitators of willingness to respond

**DOI:** 10.1186/1865-1380-5-29

**Published:** 2012-06-20

**Authors:** Chinwe Ogedegbe, Themba Nyirenda, Gary DelMoro, Edward Yamin, Joseph Feldman

**Affiliations:** 1 Emergency Trauma Department, Hackensack University Medical Center, 30 Prospect Avenue, Suite 3624, 3rd floor Main bldg, Hackensack, NJ, 07601, USA

**Keywords:** Disaster preparedness, Health care workers, Willingness to report

## Abstract

**Background:**

There is limited research on preparation of health care workers for disasters. Prior research addressed systems-level responses rather than specific institutional and individual responses.

**Methods:**

An anonymous online survey of hospital employees, who were grouped into clinical and non-clinical staff, was conducted. The objective of this study was to compare perceptions of clinical and non-clinical staff with regard to personal needs, willingness to report (WTR) to work, and level of confidence in the hospital‘s ability to protect safety and provide personal protective equipment (PPE) in the event of a disaster.

**Results:**

A total of 5,790 employees were surveyed; 41 % responded (77 % were women and 63 % were clinical staff). Seventy-nine percent either strongly or somewhat agreed that they know what to do in the event of a disaster, and the majority was willing to report for duty in the event of a disaster. The most common barriers included ‘caring for children’ (55 %) and ‘caring for pets’ (34 %). Clinical staff was significantly more likely than non-clinical staff to endorse childcare responsibilities (58.9 % vs. 48 %) and caring for pets (36 % vs. 30 %, respectively) as barriers to WTR. Older age was a significant facilitator of WTR [odds ratio (OR) 1.49, 95 % CI: 1.27-1.65]. Non-clinical staff was more confident in the hospital’s ability to protect safety and provide PPE compared to clinical staff (OR 1.43, 95 % CI: 1.15-1.78).

**Conclusion:**

Clinical and non-clinical staff differ in the types of barriers to WTR endorsed, as well as their confidence in the hospital’s ability to provide them with PPE and guarantee their safety.

## Background

Health care workers are increasingly faced with the constant threat of confronting both natural and man-made disasters. The frequency of multiple casualty incidents (MCIs), such as the September 11 World Trade Center terrorist attacks, hurricane Katrina in 2005, swine flu pandemics in 2009, and most recently the earthquake and its resultant tsunami in Northern Japan, has changed health care workers’ perspective of disaster preparedness. Despite increased public awareness of the threat of MCIs, the emphasis on preparing the US health care workforce for such disasters is inadequate [1,2]. Specifically, most of the data on disaster preparedness is based on studies of systems-level responses rather than specific institutional and individual responses. For example, in the review of responses to disaster preparedness by Chaffe et al., 90 % of the 27 studies reviewed were derived from statewide and countywide populations with only a minority from single institutions [2,3]. Kaji and colleagues in 2008 suggested that a multi-pronged approach targeted at individual health care workers and hospitals would be needed to adequately characterize overall institution preparedness [4].

Despite the existence of numerous guidelines for hospital disaster plans by several agencies (the American College of Emergency Physicians, the Joint Commission on Accreditation of Healthcare Organizations, the Department of Health and Human Services), the willingness of health care workers to report for duty in the event of a disaster varies considerably depending on the nature of the disaster [5]. For example, one of the earliest studies in disaster preparedness documented that willingness to report (WTR) for work increased in a simulated chemical attack from 42 % to 86 % if safety measures were provided [6]. In another study of 1,711 hospital workers, WTR for MCIs due to building collapse or fire was much higher (87 %) than WTR for MCIs involving a biologic agent (58 %) [7]. While numerous investigators have tried to understand the barriers to WTR [3], a key limitation of previous studies is the lack of information comparing clinical and non-clinical health care workers on “barriers to” (defined in this study as factors against) and “facilitators of” (defined in this study as contributing factors) willingness to report for duty in the event of a disaster. Furthermore, the sample sizes of existing studies were often small, making it difficult to draw valid inferences from the reported findings. Such information is sorely needed for planning purposes in the event of a disaster. A multidisciplinary approach that can take into account underlying differences in the push-and-pull factors influencing health care workers’ WTR may more likely succeed in meeting the high demand for health care services in the event of a disaster.

The objectives of this study are to compare, among a large sample of health care personnel from a community hospital, the responses of clinical and non-clinical staff with regard to their: (1) personal needs, (2) willingness to report to work in the event of a disaster, and (3) level of confidence in their hospital‘s ability to provide protective gear and take precautions to protect the their safety in the event of a disaster.

## Methods

### Study design, setting and population

The study was conducted at a 775-bed community teaching hospital in Northern New Jersey. The study design was cross-sectional, utilizing a web-based (via e-mail) survey and a paper-based (for participants without hospital e-mail access) survey. The institutional review board of the hospital approved the study. An explanatory cover letter and a 10-item questionnaire were sent to all hospital employees with e-mail access (5,600 employees) via an online link, while the same information was sent via paper-based surveys to those without hospital e-mail (190 employees). Study participants were recruited from various services/departments including security, plant operations, maintenance, emergency medical technicians (EMTs), medics, safety and nutrition. All paper-based surveys were handed out anonymously during departmental staff meetings. For the purpose of this study, the magnitude of the disaster referred to in the survey was equivalent to a code yellow level MCI, overwhelming hospital, and county or regional resources.

### Survey development

The 10-item questionnaire was designed, based on a careful review of the literature of similar studies [3,8]. Participants responded to a set of ten questions that asked about their responsibilities during a disaster (3 items); the barriers or personal needs required to fulfill their obligations (2 items); and factors that would facilitate their willingness to fulfill their responsibilities to report for duty during a disaster (5 items). Participants were also asked about their demographics and job type. With regard to barriers, they were asked to identify the personal needs that may pose barriers to their reporting for work during a disaster; for this purpose, they rated their responses on a Likert scale of 1 to 4 (1 = not an issue; 1 = somewhat easy; 2 = somewhat difficult; 3 = very difficult) to the following barriers: caring for children, caring for the elderly, caring for pets and having a second job (see Appendix). With regard to facilitators of WTR, participants were asked to identify the areas of assistance that would make it possible for them to report for duty. These included provision of transportation (to and from work), availability of childcare, availability of pet care services, home care for elderly dependents and health-related needs (e.g., filling prescriptions, doctor visits). Face validity of the 10-item questionnaire was assessed via the use of content experts (a panel of emergency management experts including three physicians) and survey methodologists to evaluate relevance of specific items and clarity of questionnaire.

### Data analysis

For demographic data, age was collapsed into two categories (<45 years and > 45 years); years of service was collapsed into four categories (≤ 1, 2–5, 6–10, >10 years); and health care workers were grouped into clinical (physicians, nurses, respiratory therapists and physical therapists) and non-clinical staff (support service employees such as plant operators, patient escorts and administrative staff). Categorical responses were compared with chi-square or Fisher’s exact test, where appropriate. Comparison of personal needs of health care workers was performed using chi-square analysis. Logistic regression was used to determine the association among willingness to report to work in the event of a disaster, participant characteristics and personal needs, whereas the factors affecting health care workers’ level of confidence in the hospital’s ability to provide PPE were examined using cumulative logistic regression models [8]. Results were presented as odds ratios (OR), their 95 % confidence interval and corresponding *p*-value. For the ordinary logistic regression model, the model fit was calibrated using the Hosmer and Lemeshow goodness of fit test [9], and for the cumulative logistic model, a Pearson’s chi-square goodness of fit test was conducted. For all tests, *P* < 0.05 was considered statistically significant. All analyses were performed using SAS 9.2 (SAS Institute Inc., Cary, NC).

## Results and discussion

## Results

The survey was administered to 5,790 hospital workers (5,600 via e-mail, 190 paper based)with a 41 % response rate for the e-mail based and 100 % response rate for the paper-based surveys. Characteristics of survey respondents are shown in Table 1. The majority were women; compared to clinical staff, the non-clinical staff was older and reported <10 years of service. More than 75 % of the study participants either strongly agreed or somewhat agreed that they know what to do in the event of a disaster; almost all survey respondents (93 %) were willing to report for duty in the event of a disaster and understood why they might be required to work overtime in the event of a disaster.

**Table 1:** Participant characteristics

**Characteristic, %**	**Clinical (*n* = 1,272)**	**Non-clinical (*n* = 750)**
Age < 45†	49.6	43.7
Female†	82.0	67.2
Years of service†		
< 1 year	4.4	6.5
2 – 5	29.1	32.6
6 – 10	28.7	36.6
>10 years	37.8	24.4

Comparison of frequencies between any groups was performed using chi-square test. †Indicates statistical significant result, *P* < 0.05.

### Comparison of health care workers on personal needs/barriers to reporting to work in a disaster

As shown in Table 2, the two most common personal responsibilities endorsed as barriers to reporting for duty in the event of a disaster were: ‘caring for children’ (endorsed by over half of the study participants) and ‘pets’ (endorsed by a third of the participants). A comparison of the personal needs and obligations of clinical and nonclinical staff is presented in Table 2. Non-clinical staff were less likely to indicate childcare responsibilities [odds ratio (OR) 0.64; (95 % CI: 0.51–0.80] and pet care (OR 0.76; 95 % CI: 0.59-0.96) as barriers to willingness to report to work in the event of a disaster. Similarly, non-clinical staff was less likely to report needing assistance for childcare (OR 0.59; 95 % CI: 0.47–0.73) and pet care (OR 0.65; 95 % CI: 0.50–0.84) in the event of a disaster. Accordingly, more clinical staff indicated on the whole that they would utilize the childcare services and pet care services if they were provided on site at the hospital.

**Table 2:** Comparison of personal needs and obligations of clinical versus nonclinical staff

**Personal needs item**	**Clinical (%)**	**Non-clinical (%)**	**OR (95 % CI)**
**I have the following responsibilities**	(*n* = 987)	(*n* = 512)	
Caring for their children†	58.9	48	0.64 (0.51-0.80)
Caring for the elderly	17.2	17.6	1.02 (0.76-1.38)
Pets†	36.1	30.1	0.76 (0.59–0.96)
Alternative/second job	16.2	15.4	0.94 (0.69–1.28)
Other	12.8	16.2	1.31 (0.95–1.80)
**I would need assistance with:**	(*n* = 1,112)	(*n* = 647)	
Child care†	39.1	27.5	0.59 (0.47–0.73)
Transportation	11.2	12.7	1.14 (0.84–1.55)
Pet care†	23.2	16.5	0.65 (0.50–0.84)
Home care for elderly	8.5	10.2	1.21 (0.86–1.71)
Home-related needs	7.6	6.2	0.79 (0.52–1.19)
N/A†	34.0	43.4	1.49 (1.21–1.82)
**I would utilize services offered by the hospital:**	(*n* = 1,097)	(*n* = 656)	
Child care (onsite) †	25.8	20.7	0.75 (0.59–0.95)
Transportation	21.0	22.9	1.11 (0.87–1.41)
Pet care†	18.9	12.4	0.60 (0.45–0.80)
Home care for elderly	7.8	7.8	0.99 (0.67–1.44)
Home-related needs	8.2	7.9	0.97 (0.66–1.41)
Other	3.5	4.4	1.28 (0.75–2.17)

†Indicates a significant result, *P* < 0.05. CI, confidence interval.

### Barriers and facilitators of health care workers’ willingness to report to work

Table 3 shows unadjusted and adjusted multivariable analysis of predictors of willingness of health care workers to report to work in the event of a disaster. With regard to participant characteristics, only age emerged as a significant facilitator of WTR. Specifically, as the age group increased, the more likely the health care worker was willing to report to work (OR 1.49; 95 % CI: 1.27-1.65). Gender, years of service and job type were not related to willingness of the health care worker to report to work in the event of a disaster. With regard to personal needs, eldercare emerged as a significant barrier such that those with eldercare obligations were less likely to be willing to report to work compared to those without such obligations (OR 0.58, 95 % CI: 0.36-0.94). Job type was unrelated to employees’ willingness to report for work in the event of a disaster (OR 0.71; 95 % CI: 0.46 to 1.11).

**Table 3:** Association between willingness to report to work in the event of a disaster and participant characteristics and personal needs/obligations

	**Unadjusted odds ratio (OR)**		**Adjusted odds ratio (OR)**	
Characteristic: ref. category	OR (95 % CI)	*P*-value	OR (95 % CI)	P-value
Age group^b^: <45 years	1.49 (1.27–1.65)	<0.001	--	--
Gender: male	0.93 (0.60 – 1.44)	0.74	--	--
Years of service	1.16 (0.95 – 1.43)	0.15	--	--
Type of job: clinical	0.72 (0.50 – 1.03)	0.07	0.71 (0.46 – 1.11)	0.13
Childcare: no	0.91 (0.60 – 1.37)	0.64	--	--
Eldercare: no	0.58 (0.36 – 0.94)	0.02	--	--
Pet care: no	1.01 (0.66 – 1.55)	0.95	--	--
Alternative/second	0.78 (0.47 – 1.32)	0.35	--	--

ref = reference, CI = confidence interval, ^b^age group: <45; ≥45; ^d^years of service class: <1; 2–5; 6–10;10+. Clinical: clinical = (nursing, other clinical, physician), nonclinical = (fiscal/administrative, support staff). The odds ratio for willingness to report to work among the types of jobs was adjusted for age group, gender, years of service, type of job, childcare, eldercare, pet care and alternative/second job. †Indicates a significant predictor, *P* < 0.05. Multivariable logistic regression model yielded an adequate fit (Hosmer-Lemeshow test chi-square = 11.3, *P* = 0.185).

### Comparison of the level of confidence of health care workers in the hospital’s ability to provide personal protective equipment (PPE) and take precautions to protect their safety

Almost all study participants were either very confident or somewhat confident that the hospital will provide them protective gear and protect their safety during a disaster (94 %). Analysis of the relationship among the participants’ demographics, their job type and their confidence in the hospital’s ability to provide PPE was performed using the response variable confidence, which had three ordinal levels: very confident, somewhat confident and not confident at all. Thus, for this categorical variable with multiple responses, a cumulative logistic regression model was used to model odds of being more confident to less confident. In addition to assessing goodness of fit of the model, the proportional odds assumption required in such model was validated. When latter assumption was violated, the responses were suitably dichotomized. In this investigation, the proportional odds assumption was not satisfied in modeling confidence of the HCWs on their gender. As shown in Table 4, non-clinical staff had 1.43 greater odds of being more confident in the hospital’s ability to provide PPE and safety precautions compared to clinical staff (OR 1.43; 95 % CI: 1.15-1.78).

**Table 4:** Factors affecting HCWs’ confidence in the hospital’s ability to provide protective gear and take precautions to protect their safety

	**Unadjusted odds ratio (OR)**		**Adjusted odds ratio (OR)**	
Characteristic: ref. category	OR (95 % CI)	P-value	OR (95 % CI)	*P*-value
Age group^b^: ≥ 45 years	0.70 (0.60 – 0.83)	<0.001	--	--
**Gender: male**				
Model 1	0.97 (0.79 – 1.19)	0.78	--	--
Model 2	0.52 (0.35 – 0.75)	<0.001	--	--
Years of service: < 1	--	0.06	--	--
Type of job: clinical	1.40 (1.17 – 1.67)	<0.001	1.43 (1.15 – 1.78)	0.01

Ref = reference, CI = confidence interval, ^b^age group: <45; ≥45; ^d^years of service class: <1; 2–5; 6–10; 10+. Clinical: clinical = (nursing, other clinical, physician), Nonclinical = (fiscal/administrative, support staff). The odds ratio for levels of confidence in the hospital’s ability to provide the protective gear among the types of jobs was adjusted for age group, gender, years of service, type of job, childcare, eldercare, pet care and alternative/second job. †Indicates a significant predictor, *P* < 0.05. Multivariable cumulative logistic regression model fit was adequate with a proportional odds test: chi-square = 9.08, *P* = 0.247, Pearson goodness-of-fit test: chi-square = 0.95, *P* = 0.668.

**Figure 1:**
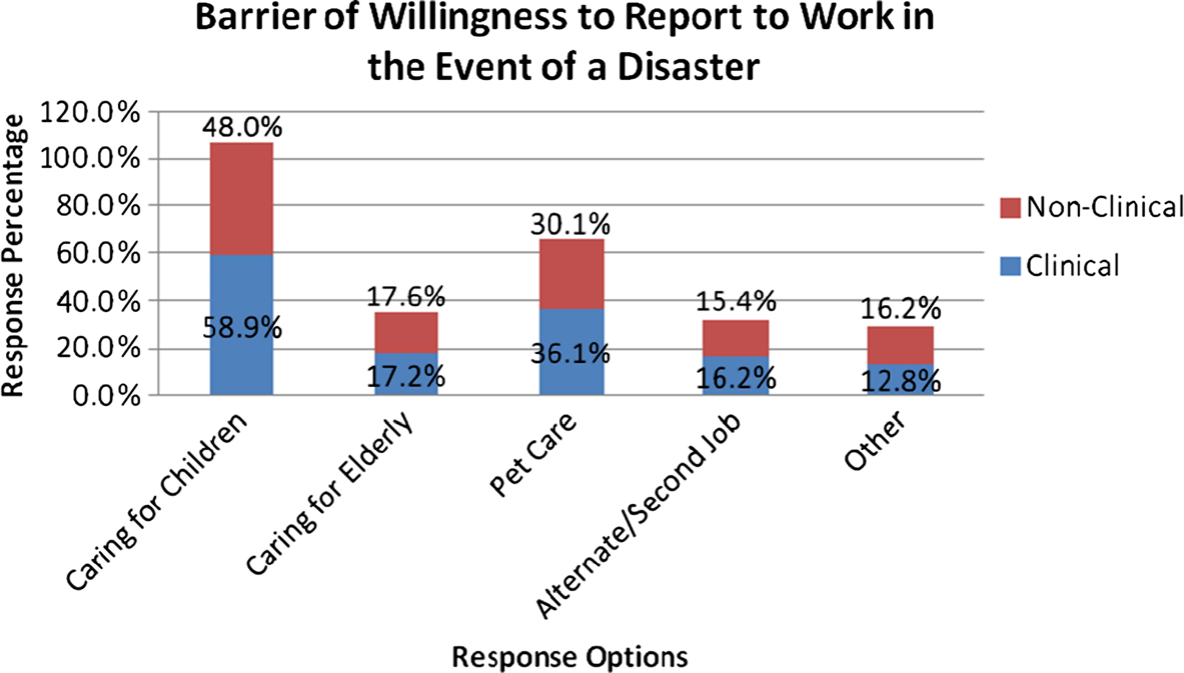
Demonstrates favorable time frames selected by clinical and non clinical staff.

With regard to a survey question that states “I can leave work, take care of my personal needs and return to work within 1 h, 2 h, or 3 h and longer,” the majority (59 %) that answered that question indicated they would be able to do so within 2 h, 3 h and longer (see Figure 1). The majority of the employees indicated that in case of a disaster, they needed to leave work to take care of personal obligations before returning to work. Comparison of non-clinical versus clinical staff in this regard indicated that over a third of the non-clinical staff did not need to leave work compared to a quarter of the clinical staff. Conversely, 33 % of the clinical staff needed 3 h or longer to be away from work in order to take care of personal obligations in the event of a disaster, and then report to work compared to only 25 % of the non-clinical staff (*P* < 0.0001).

The study has the following limitations. First, there were few physicians in our study sample, making our findings not generalizable to physicians. Second was the low response rate of 41 % despite three reminder e-mails and extension of survey deadline by 14 days. Compared to existing studies, our response rate in this largely web-based survey was higher (41 %) than that reported for another web-based study of disaster preparedness (28 %) [10]. While this rate may seem low, the range falls well within that noted in the review by Chaffe, which ranged from 28 %-61 %, with only 3 of 27 articles reviewed reporting a 100 % response rate. We should note that our survey was mostly administered via web-based questionnaire, which traditionally has a low response rate compared to paper-based questionnaires. Our paper-based survey had a response rate of over 90 %. Third, our survey utilized a convenience sample; thus, the respondents were possibly those who had strong feelings about disaster preparedness. In order to mitigate this issue, we compared responders to non-responders on important demographic characteristics and found that they were similar. Another important limitation was that though we found that older employees were mostly nonclinical and more likely to volunteer in a disaster, we did not compare older nonclinical with older clinical with regards to WTR. Finally, as is typical for this kind of work and similar to other studies, we cannot speculate on whether or not the intention to respond to disaster actually reflects real-world behavior in the event of a disaster.

## Discussion

Among 2,351 hospital workers from a community teaching hospital, we evaluated the barriers to and facilitators of willingness to report for work in the event of a disaster. Although almost all the study participants understood their responsibilities and were willing to report to work in a disaster, the majority voiced significant barriers to such activities. The most common barriers identified were personal responsibilities of caring for children, the elderly and pets. Clinical staff endorsed more barriers than non-clinical staff, and they were equally more likely than non-clinical staff to endorse their willingness to utilize hospital-based services for pet care and childcare if provided by the hospital. Conversely, the only facilitator of WTR was older age, whereas gender, years of service and job type did not make a difference. Because participants were divided into clinical and non-clinical staff, obtaining a significant difference with respect to WTR would have been a significant finding, similar to results reported by others, e.g., Qureshi et al. [2]. We however reported a null result on job type (clinical vs. non-clinical staff), and this finding may be a reflection of the low number of physicians in our survey. Finally, almost all the workers, regardless of the job type, were confident in the hospital’s ability to provide PPE and protect their safety in the event of a disaster, with non-clinical staff reporting greater odds of confidence in the hospital’s ability to provide them PPE. Similarly, clinical staff needed 3 h or longer to leave work in order to take care of personal obligations in the event of a disaster compared to non-clinical staff.

Our findings mirror those of other studies, which also found that the influence of family responsibilities and concerns for personal safety were important predictors of WTR for duty in the event of a disaster [2,11-16]. A previous study that surveyed 47 health care facilities, with a variety of institution type and volume in a metropolitan region, found that health care workers were most able to report to work for MCIs, followed closely by natural disasters, then disasters involving certain biological agents and least was for disasters involving a radiological event [5]. Unlike other studies, however, we found that needs of the participants coincided with the facilitators endorsed. For example, emergency assistance with childcare was listed as a personal need, while availability of such assistance from the hospital was endorsed as a facilitator.

Our study is one of few to report a large number of participants with knowledge of their responsibilities in the event of a disaster. A major reason that a large majority of respondents in our study (93 %) considered it their responsibility to report to work in the event of a disaster was probably based on the fact that our hospital employees had had frequent exposure to disaster preparedness training. Immediately post September11 2001, our institution put in place an elaborate hospital-wide emergency management committee, a disaster committee that was responsible for organizing regular hospital-wide disaster drills and educational sessions through the various departments.

The following strengths of our study should be noted. First, the sample size was large, and in comparison to other studies, our study has the highest number of participants in any single institution. Second, in addition to identifying the barriers to the willingness of health care professionals to work, we also identified facilitators of their willingness to work in the event of a disaster. This is an important addition to the literature in that it provides initial data needed for the development of educational interventions targeted at improving hospital employees’ WTR for duty in the event of a disaster. Third, the study participants in our study, unlike most in the more recent literature, included, in addition to clinical staff, other health care workers who perform vital services, including support services (plant operations, patient escort services and others), fiscal, information technology and human resources. This allowed us to compare the responses from these different groups of employees, considering them in clinical versus nonclinical perspective. Such comparisons are crucial given the importance of multidisciplinary strategies targeted at addressing disasters. Furthermore, educational interventions can be developed based on these responses targeted at non-clinical staff versus the clinical staff. It is vital to understand how personnel may make decisions when faced with competing priorities so that plans, policies and organizational decisions can be based on the best evidence available [3].

## Conclusions

In summary, there are few data on the perceptions of WTR among health care workers in individual institutions. In this study from a community teaching hospital, we reported various barriers and facilitators of WTR in the event of a disaster. More importantly, we found that clinical and non-clinical staff differed in the types of barriers to WTR endorsed as well as their confidence in the hospital’s ability to provide them with PPE and guarantee their safety. These findings underscore the importance of conducting surveys at the individual institution level rather than adopting only statewide or countywide approaches to addressing disaster preparedness of the hospital workforce. This is an important finding, given that individual hospitals will have barriers that are specific to the needs of their employees. Future studies should evaluate disaster preparedness among hospital workers in individual institutions rather than across counties and statewide health systems. Such research would provide crucial information for planning interventions that address institution-level factors. Furthermore, the effect of institution-wide training of clinical and non-clinical staff on disaster preparedness should be evaluated in future studies. Such training should emphasize employee responsibility, as well as highlight the institutions’ concerns and plans for the highest level of employee safety.

## Appendix

### Appendix: Web based Questionnaire fielded for this study

1. Employee Responsibilities During a Disaster

1. As a member of my department, I know what to do in the event of a disaster.

 Strongly disagree

 Somewhat disagree

 Somewhat agree

 Strongly agree

2. **If a disaster occurs, I understand why I may be asked to stay past my shift to assist the medical center.**

 Yes

 No

3. **If the hospital is in a disaster situation, I will report to work as scheduled.**

 Yes

 No

2. **Personal Needs**

1. **I have the following personal responsibilities that may prevent me from working past my shift or reporting to work, outside of my normal schedule, in the event of a disaster. (Check all that apply)**

 Caring for children

 Caring for the elderly

 Pets

 Alternate/second job

 Other

If Other, please specify:

2. **In order to fulfill my obligation to report to work past my shift or report to work outside of my normal schedule, I would need assistance with:**

 Child Care

 Transportation (rides to/from work; carpooling, etc.)

 Pet Care

 Home care for elderly parents

 Health-related needs (i.e. filling prescriptions, doctor visits, etc)

 N/A

 Other

If Other, please specify:

3. **In order to fulfill my obligation to work outside of my normal schedule, I would utilize the following services offered by HUMC if available:**

 Child Care (on-site)

 Transportation (rides to/from work; carpooling, etc.)

 Pet Care (dog walking, feeding, pet sitting)

 Home care for elderly parents

 Health-related needs (i.e. filling prescriptions, doctor visits, etc)

 Other

 I would not utilize services offered by HUMC

If Other, please specify:

4. **Arranging for dependent care (child/elder/pet) in case I need to be away from home for several days will be:**

 Very difficult

 Somewhat difficult

 Somewhat easy

 Not an issue

5. **I can leave work and take care of my personal needs and return back to work within:**

 I do not need to leave work

 1 hour

 2 hours

 3 hours or longer

6. **Having crisis counselors available to me at HUMC in the event of a disaster is:**

 Not important to me

 Somewhat important

 Very important

 Essential

7. **My level of confidence that HUMC will provide me with protective gear and take precautions to protect my safety during a disaster (if the situation requires it)**

 Not confident at all

 Somewhat confident

 Very confident

3. **Demographics**

1. **Years of service with HUMC:**

 Less than 1

 2–5

 6–10

 11–15

 16–20

 20+

2. **Your age:**

 18–25

 26–34

 35–44

 45–54

 55–64

 65+

3. **Your gender:**

 Male

 Female

4. **Please select the division you work in**

 Nursing

 Other Clinical (i.e. Respiratory, Physical Therapy, etc)

 Physician

 Support Services (i.e. Plant Operations, EVS, NFM, Patient Escort, etc)

 Fiscal and Administrative (i.e. Finance, HR, IT, HIM, etc)

5. **Please select the following list of choices that best describes your discipline**

 Administrative Professional/Secretary

 Physician (Attending)

 Case Management

 Consumer Affairs/Guest Services

 Diagnostic Technician

 Dietary Services

 Distribution

 Environmental Services

 Finance

 Health Information

 Human Resources

 Nursing

 Nursing Assistant

 Pathology

 Patient Escort

 Pharmacy

 Physical Therapy

 Physician

 Plant Operations

 Purchasing

 Radiology

 Physician (Resident)

 Respitatory Therapy

 Security

 Social Services

 Unit Clerk/Coordinator

 Volunteer

 Other

If Other, please specify:

## Abbreviations

HCW, health care workers; WTR, willingness to report; PPE, personal protective equipment; MCI, multiple casualty incident; EMT, emergency medical technician.

## Competing interests

The authors declare that they have no competing interests.

## Authors’ contribution

CO conceived the study, designed the survey instrument, drafted the manuscript and interpreted the data. JF obtained the research funding, conceived the study and supervised the manuscript preparation. GD supervised the conduct of the study and supervised the administration of the survey. TN carried out the data management and data analysis, and participated in interpretation of the data. EY was responsible for recruiting participants and the study design. All authors contributed substantially to its revision. CO takes responsibility for the paper as a whole. All authors read and approved the final manuscript.

## Authors’ information

EY (Board-certified staff ED physician; Vice Chair, Emergency Department, lead physician in charge of the disaster program of the institution, coordinates regular disaster drills and liaises with hospital leadership in disaster operational plans), TN (PhD statistician, Department of Research, Hackensack UMC), GD (Manager for Disaster Program at Hackensack UMC, runs all disaster drills, coordinates all disaster management efforts in the institution), JF (Chairman and ED Physician at Hackensack UMC, in charge of all Emergency Services in the institution), CO (Section Chief for research and ED Physician at Hackensack UMC, in charge of all research activities in the ED, takes overall responsibility for this manuscript submission).
